# Ovarian dysgerminoma and synchronic contralateral tubal pregnancy followed by normal intra-uterine gestation: a case report

**DOI:** 10.1186/1752-1947-6-399

**Published:** 2012-11-23

**Authors:** Lourdes Montesinos, Pedro Acién, Monserrat Martínez-Beltrán, María-José Mayol

**Affiliations:** 1 Service of Obstetrics and Gynecology, San Juan University Hospital, Alicante, Spain; 2 Service of Pathology, San Juan University Hospital, Alicante, Spain; 3 Departamento/Area de Ginecología, Facultad de Medicina de la Universidad “Miguel Hernndez”, Campus de San Juan, Alicante, 03550, Spain

**Keywords:** Dysgerminoma, Ovarian tumors, Germ cell tumors, Ectopic pregnancy, β-hCG, LDH, Pregnancy after surgery

## Abstract

**Introduction:**

We report that the coincidence of ovarian tumor and pregnancy poses significant challenges that are more pronounced if the pregnancy is ectopic.

**Case presentation:**

Here, we report a rare and interesting case of a 24-year-old nulliparous Spanish woman who experienced the coincidental occurrence of left tubal pregnancy and dysgerminoma in the right ovary. The corpus luteum settled in the right ovary. A right adnexectomy and left linear salpingostomy were performed. Remarkably, our patient became pregnant spontaneously after surgery. The pregnancy occurred prior to starting chemotherapy, and the intra-uterine pregnancy was carried to term; later, she also had another normal pregnancy. Our patient has done well without chemotherapy.

**Conclusions:**

Our report on the challenges of diagnosis and treatment faced in this case can help clinicians better understand and manage these pathologies. We have not found any similar cases in the literature.

## Introduction

The coincidence of an ovarian tumor and a pregnancy poses significant challenges for differential diagnosis, which are more pronounced if the pregnancy is ectopic. In cases of ectopic pregnancy, a positive human β-chorionic gonadotropin (β-hCG) test result, absence of a visible intra-uterine gestation upon ultrasound examination and presence of an ovarian tumor also suggest a germ cell tumor. We present the exceptional case of a patient who experienced the coincidental occurrence of a tubal ectopic pregnancy with a large tumor in the contralateral ovary that was revealed to be a dysgerminoma. Interestingly, our patient became pregnant after undergoing laparotomy for a right adnexectomy and a left linear salpingostomy. Our patient carried this pregnancy to term and later had another normal intra-uterine pregnancy that was also carried to term.

## Case presentation

A 24-year-old nulliparous Spanish woman first presented seven years ago to the obstetric-gynecologic emergency service (OGES) of our hospital with a history of pelvic pain, weight loss, metrorrhagia after menstrual delay, urinary infection, positive β-hCG test result and the finding of a right ovarian tumor upon examination.

She underwent menarche at age nine, had frequent menstrual delays and had been taking oral contraceptives (OC) since age 17. She had a history of tobacco and drug (not parenteral) use. Prior to her presentation, she had not undergone regular gynecological exams but had presented several times to the OGES. Two years earlier, she presented after two months of amenorrhea. A pregnancy test was negative, and a transvaginal ultrasound examination (TVU) showed a 2cm anechoic tri-chamber image in the right ovary. Her blood sedimentation rate (BSR) was normal at 14mm (normal value (nv), <25mm), and the following tests for tumor markers were negative: β-hCG <1.2mU/mL (nv, 0 to 5mU/mL); cancer antigen (CA)-19-9 15.87U/mL (nv, 0 to 40U/mL); carcinoembryonic antigen (CEA) 0.97ng/mL (nv, 0 to 5ng/mL); CA-125 14.53U/mL (nv, 0 to 35U/mL); α-fetoprotein (α-FP) 1.05ng/mL (nv, 0 to 15ng/mL). Our patient returned to the OGES for pelvic pain and urinary symptoms six months later. The results of the TVU exam and the blood work were similar to those from our patient’s previous presentation (BSR 17mm, CA-19-9 17.76U/mL, CEA 0.95ng/mL, CA-125 12.23U/mL). Again, our patient did not attend for subsequent outpatient follow-up.

**Figure 1:**
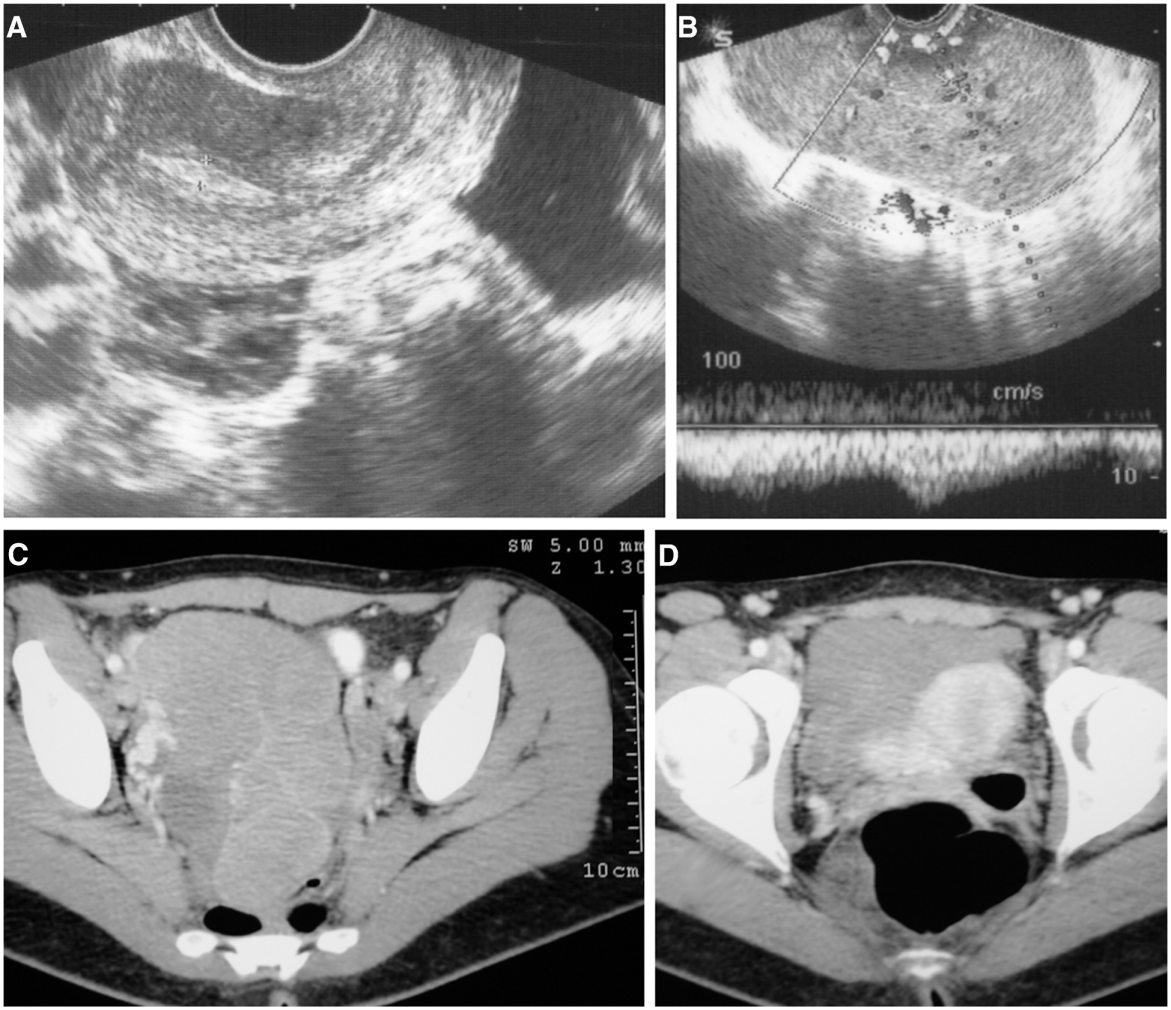
**Imaging studies.** (**A**) Uterus and hemoperitoneum transvaginal ultrasound images. (**B**) Right ovarian tumor and Doppler ultrasonography showing a diffuse vascular mapping with low resistance index. (**C**) Right ovarian tumor on pelvic computed tomography scan. (**D**) Tumor and uterus on pelvic computed tomography scan.

When she returned to OGES seven years ago she presented with persistent metrorrhagia and symptoms of a urinary infection, as mentioned above. A pregnancy test was weakly positive and an altered urinary sediment confirmed urinary tract infection. A TVU did not reveal an intra-uterine pregnancy, but a right adnexal hyperechoic dotted mass was observed. It appeared to be heterogeneous and of medium density and measured 7×6cm. Our patient underwent repeat blood analysis and her tumor markers were as follows: BSR 29mm, β-hCG 105U/mL, CA-19-9 15.34U/mL, CEA 0.99ng/mL, CA-125 13.73U/mL, α-FP 1.06ng/mL. She was told to take urinary antibiotics, repeat the analysis and return to our outpatient center two weeks later. She reported experiencing weight loss and minor metrorrhagia for one month and had discontinued her OC four months prior to her visit. A vaginal examination showed a normal uterus and a large right adnexal tumor extending from the Douglas cul-de-sac to the right iliac fossa. A TVU exam showed a normal uterus with no evidence of endometrial pathology, a normal left ovary, low to moderate ascites and a large pelvic tumor. The tumor measured over 13cm, was poly-lobed and had heterogeneous densities with increased vascularity, arising from the right ovary. An endometrial biopsy was performed and revealed secretory changes without villi or trophoblastic elements. Repeated blood tests showed the following: a normal hemogram, BSR 8mm and normal blood chemistry with the exception of lactate dehydrogenase (LDH) 2349U/L (nv, 135 to 214U/L) and β-hCG 242U/mL; α-FP, CA-19-9, CEA and CA-125 levels were normal. A chest radiograph was normal, and abdominal and pelvic computed tomography (CT) and Doppler sonography showed a solid right ovarian mass of 13×8cm with diffuse vascular mapping with low resistance index and fluid in the Douglas cul-de-sac (Figure 1).

**Figure 2:**
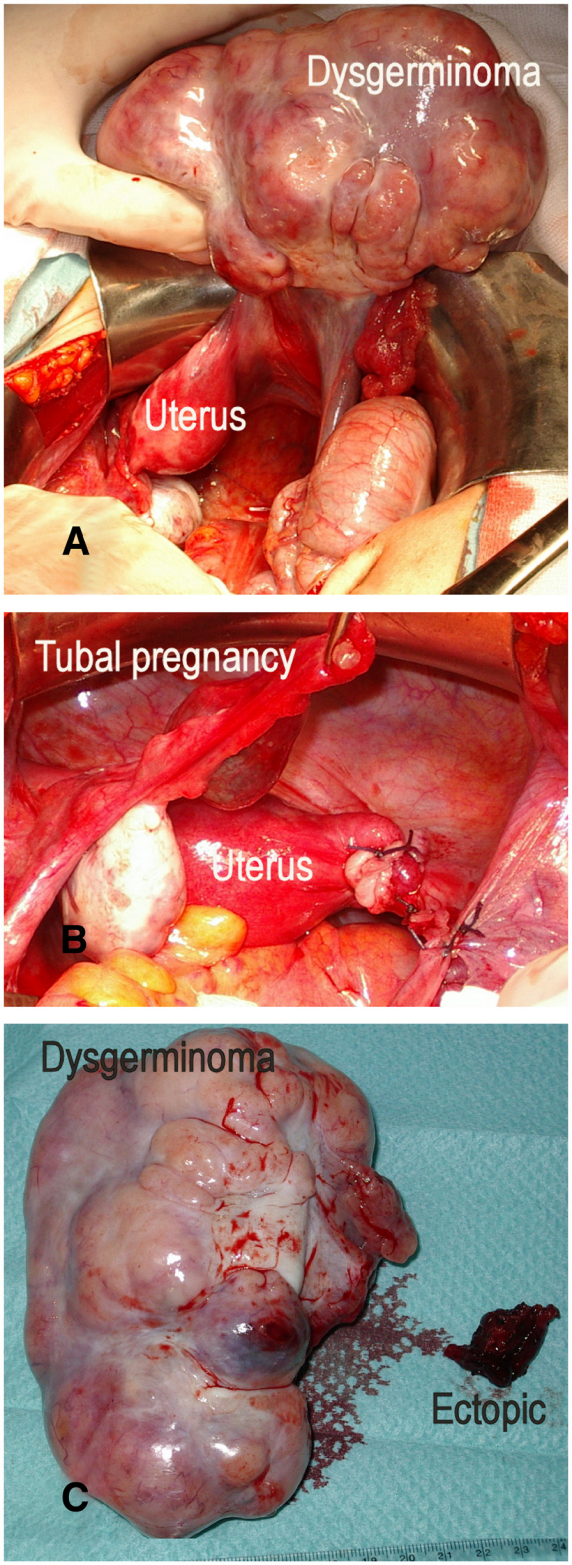
(**A**) **Laparotomic observation.** Right ovarian tumor and uterus. (**B**) After right adnexectomy: left tubal pregnancy, left ovary and uterus. (**C**) Ovarian tumor and tissue from ectopic pregnancy.

With the diagnosis of a right ovarian tumor compatible with a germ cell tumor, which was thought likely to be a dysgerminoma, laparotomy was performed three weeks later. During the laparotomy procedure, we found a solid, poly-lobed and irregular right ovarian mass of roughly 14cm in size, as well as an ectopic pregnancy in the ampullar region of the left tube (Figure 2). There was a small amount of hemoperitoneum, approximately 50 to 100cm^3^. A right adnexectomy and a left linear salpingostomy were performed. Her postoperative course was uncomplicated and she had menstruation after surgery.

**Figure 3:**
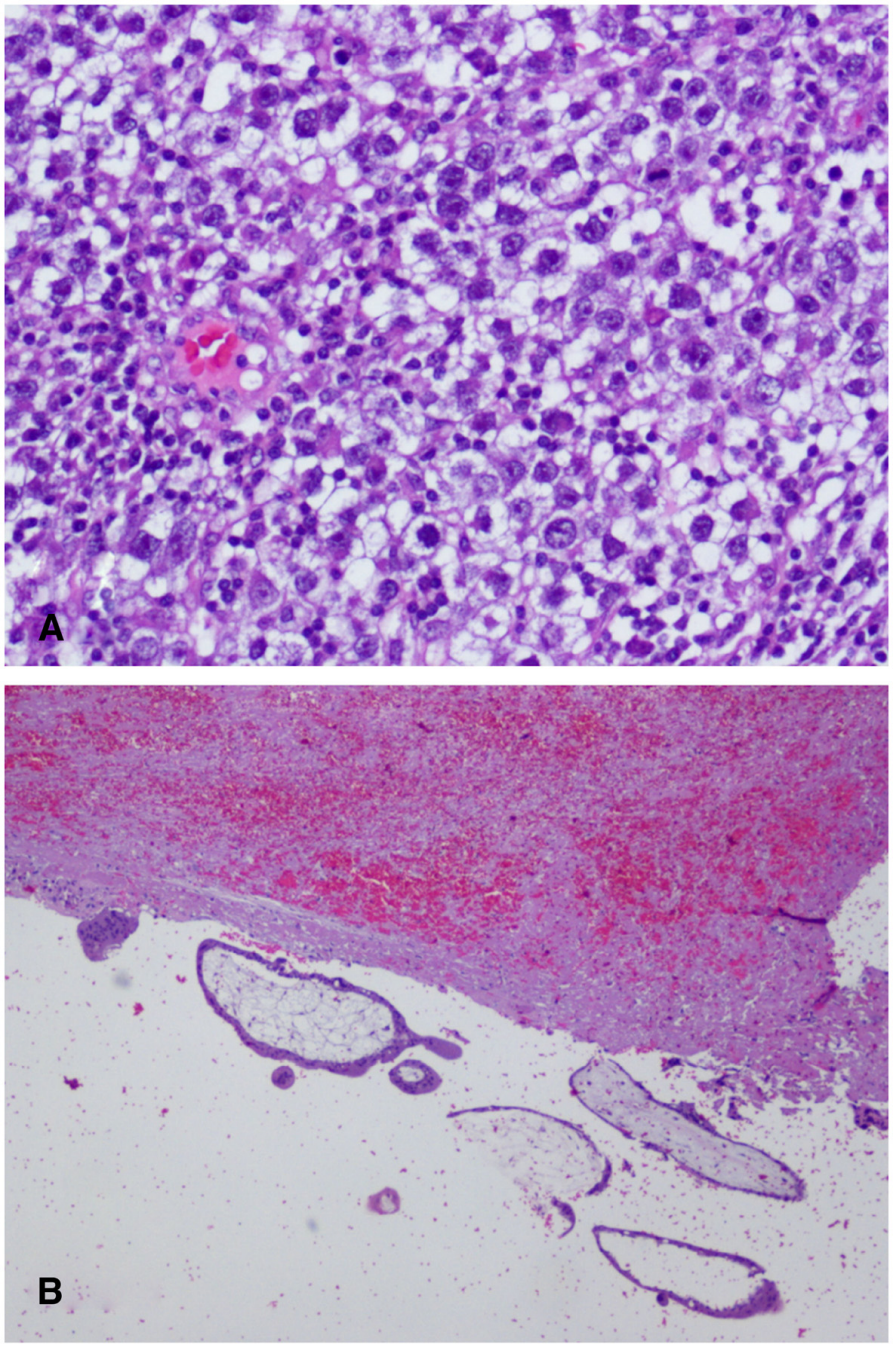
**Histopathological images.** (**A**) Dysgerminoma, 20×, hematoxylin and eosin stain. (**B**) Chorial villi tissue and fibrinohemorrhagic material, 4×, hematoxylin and eosin stain.

The histopathologic diagnosis was as follows: (1) left tubal ectopic pregnancy; (2) negative results for peritoneal fluid cytology; and (3) right ovarian dysgerminoma 12cm in size, with areas of tumor necrosis and infiltration of the tunica albuginea but with an unruptured capsule. Histopathological images are shown in Figure 3. A normal residual ovary was identified with the presence of the corpus luteum. Immunohistochemical analysis gave the following results: keratin was weakly positive; desmin, vimentin, and neuron specific enolase were all negative; and the proliferative index was high (>50 percent). The right fallopian tube had a normal appearance. On absence of peritoneal tumor spread on thorough inspection, the International Federation of Gynecology and Obstetrics (FIGO) stage was likely consistent with a stage IA.

After discussing the case at our Gynecological Tumors committee, it was decided to administer three chemotherapy cycles in spite of likely stage IA dysgerminoma due to no biopsy of pelvic and para-aortic lymph nodes, with our patient transferred to the medical oncology service. However, a new positive pregnancy test postponed the start of chemotherapy. Our patient returned to the OGES five weeks after surgery because she had experienced no menstruation, her β-hCG was 588U/mL, and a TVU confirmed the presence of an early intra-uterine pregnancy. Our patient was informed of the situation, and she decided to continue the pregnancy. A new TVU confirmed the presence of a normal intra-uterine pregnancy of gestation five to six weeks, with a normal yolk sac and embryo button. Four weeks later the embryo measured 24mm, which is consistent with a gestational age of roughly nine weeks, indicating that our patient became pregnant less than three weeks after surgery. The pregnancy evolution was normal, although there was some delay of fetal growth at the end of gestation. Our patient gave birth to a baby boy, born at term with a weight of 2600g. Chemotherapy was subsequently dismissed.

Our patient moved to another city and again became pregnant. She delivered one year later at the Baza Hospital via Caesarean section due to intra-uterine growth retardation and fetal distress. From that point to the present day, our patient has been followed up in our hospital Oncology and Gynecology departments. She remained asymptomatic and with normal test results (β-hCG negative; LDH = 302U/L in 2009, 155U/L in 2010 and 150 in 2011 and 2012; α-FP and other tumor markers were all normal) and normal imaging studies (TVU and CT).

## Discussion

Dysgerminomas are tumors derived from germ cells, which constitute 3 to 5 percent of malignant ovarian tumors. They are rare tumors that affect younger patients, and treatment should therefore be as unaggressive as possible and aim to preserve fertility. Most dysgerminomas are unilateral and occur predominantly on the right side, but up to 12 percent of cases may be bilateral [1]. The combination of conservative surgery and chemotherapy achieves good results even in advanced stage disease. In the early stages, it is generally recommended that the patient undergo conservative treatment (unilateral salpingo-oophorectomy; USO) and platinum-based adjuvant chemotherapy. However, with stage IA tumors treatment with surgery alone is likely sufficient. Nevertheless, dysgerminoma of the ovary has a propensity to metastasize to the pelvic and para-aortic lymph nodes in the absence of other evidence of metastatic disease; biopsies of these structures are particularly important [2]. In a recent review of dysgerminomas of the ovary, Vicus et al. [3] reported that from 65 cases in their institution during a period of 35 years, 38 (58.5 percent) cases presented with stage IA disease and in five of them (13.1 percent) recurrences occurred after performing USO (unstaged), all within 19 months of the primary diagnosis. However, none of the 15 patients who received adjuvant treatment experienced tunorrecurrence, and three women who had received adjuvant chemotherapy had successful pregnancies. Shamim [4] also reported a case of a 30-year-old woman presenting with a successful pregnancy outcome after fertility-sparing surgery and chemotherapy for dysgerminoma. Our patient has done well without adjuvant treatment.

Diagnosing ovarian germ cell tumors is challenging [5]. Following clinical examination and imaging tests (ultrasound, CT and magnetic resonance imaging (MRI)), the finding of increased serum levels of β-hCG, α-FP and LDH suggest the diagnosis pre-operatively. Although there is considerable variation in the production of these markers, the majority of endodermal sinus tumors produce α-FP, and most choriocarcinomas and dysgerminomas produce β-hCG and LDH respectively. However, some dysgerminomas also produce β-hCG [1].

The case presented here had the unusual coincidence of being associated with a tubal pregnancy, which increased β-hCG and provoked a slight hemoperitoneum, thus raising doubts about the diagnosis. In fact, once the ovarian tumor had been diagnosed, the tubal ectopic pregnancy was not suspected and was only found at the time of surgery. Interestingly, the corpus luteum was present in the right ovary, as was the dysgerminoma, while the ectopic pregnancy was implanted in the contralateral tube. It is possible that the tumor favored the ectopic pregnancy on the contralateral side (external hyperemigration). In the literature, we have found few cases of tubal pregnancy associated with ovarian carcinoma and only one associated with dysgerminoma [6]. This case concerned a patient who presented with a dysgerminoma in the left ovary and a right interstitial pregnancy; a corpus luteum was present in the ovary with the dysgerminoma. An emergency laparotomy was performed due to accidental breakage of the ectopic pregnancy with acute hemoperitoneum. The treating clinicians performed left adnexectomy and cuneiform resection of the right uterine horn with salpingectomy. The authors reviewed the literature and did not find any other similar cases. We were also unable to find any similar published cases at present.

There are cases of gonadoblastoma and tubal pregnancy [7,8], and there is a series of 23 cases of ovarian carcinoma during pregnancy from Dgani et al. [9], including four dysgerminomas at stage IA associated to intra-uterine gestation. There were three cases of ovarian carcinoma and right tubal pregnancy, but two were serous carcinomas and one was a granulosa tumor; all three patients had advanced illness.

In our patient’s case, it is also interesting that after the right adnexectomy and the left salpingostomy, which were performed during laparotomy, our patient became pregnant with a normal intra-uterine pregnancy and carried it to term; she later also had another normal pregnancy. It is clear that despite the surgery that was performed, our patient’s fertility was effectively preserved. However, our report on the challenges of diagnosis and treatment in this case can help clinicians to better understand and manage these pathologies.

## Conclusions

We report the interesting case of a 24-year-old nulligravid woman who experienced the coincidence of left ectopic pregnancy and dysgerminoma in the right ovary. This raised important diagnostic challenges in view of the observation of a positive β-hCG test without detecting the ectopic pregnancy. A laparotomy with right adnexectomy and left linear salpingostomy were performed. Remarkably, our patient became pregnant spontaneously after surgery. The pregnancy occurred prior to starting chemotherapy, and the intra-uterine pregnancy was carried to term; later she also had another normal pregnancy. Our patient has done well without chemotherapy. We have not found any similar cases in the literature.

## Consent

Written informed consent was obtained from the patient for publication of this case report and any accompanying images. A copy of the written consent is available for review by the Editor-in-Chief of this Journal on request.

## Competing interests

The authors declare that there are no conflicts of interests.

## Authors’ contributions

LM reviewed the case, participated in the study and surgical operation and reviewed the manuscript. PA studied our patient, designed the study, reviewed the literature, created the figures and wrote the paper. MMB participated in the study and surgical operation and reviewed the manuscript. MJM performed the histopathological studies. PA had full access to all of the data in the study and takes responsibility for the integrity of the data and the accuracy of the data analysis. All authors read and approved the final manuscript.

## References

[B1] The American College of Obstetricians and Gynecologists (ACOG) Precis V, An Update in Obstetrics and Gynecology, 1998. Edición Española: Actualización en Obstetricia y Ginecología Cancer de Ovario y de Trompa 1999 Editado por Medical Trends, SL 328 339

[B2] Baker VV DeCherney AH, Pernoll ML Premalignant and malignant disorders of the ovaries and oviducts Current Obstetrics and Gynaecologic Diagnosis and Treatment 1994 Eighth Appleton & Lange. Prentice-Hall International, Inc 964 984

[B3] Vicus D Beiner ME Klachook S Le LW Laframboise S Mackay H Pure dysgerminoma of the ovary 35 years on: a single institutional experience Gynecol Oncol 2010 117 23 26 10.1016/j.ygyno.2009.12.024 20097412

[B4] Shamim M Successful pregnancy outcome after fertility-sparing surgery and chemotherapy for dysgerminoma J Pak Med Assoc 2010 60 779 781 21381594

[B5] Lazebnik N Balog A Bennett S Redline R Liu J Ovarian dysgerminoma: a challenging clinical and sonographic diagnosis J Ultrasound Med 2009 28 1409 1415 19778893 10.7863/jum.2009.28.10.1409

[B6] Ribeiro ER Marcondes-Fonseca N Dysgerminoma of the ovary and interstitial pregnancy (article in Portuguese) An Bras Ginecol 1964 58 1 8 14206206

[B7] Pratt-Thomas HR Cooper JM Gonadoblastoma with tubal pregnancy Am J Clin Pathol 1976 65 121 225 942810 10.1093/ajcp/65.1.121

[B8] Elemenoglou J Korkolopoulou P Zizi A Delides GS A case of gonadoblastoma in a normal woman with tubal pregnancy Arch Geschwulstfotsch 1990 60 223 226 2369284

[B9] Dgani R Shoham Z Atar E Zosmer A Lancet M Ovarian carcinoma during pregnancy: a study of 23 cases in Israel between the years 1960 and 1984 Gynecol Oncol 1989 33 326 331 10.1016/0090-8258(89)90521-0 2722058

